# Apparent lack of spill-over of parasites from an invasive anuran: PCR detects *Entamoeba* in cane toads (*Rhinella marina*) but not in sympatric Australian native frogs

**DOI:** 10.1016/j.ijppaw.2020.06.009

**Published:** 2020-06-30

**Authors:** Phoebe Rivory, Gregory Brown, Cathy Shilton, Richard Shine, Jan Šlapeta

**Affiliations:** a Sydney School of Veterinary Science, Faculty of Science, University of Sydney, New South Wales, 2006, Australia; b Department of Biological Sciences, Faculty of Science and Engineering, Macquarie University, New South Wales, 2109, Australia; c Berrimah Veterinary Laboratory, Northern Territory Government, GPO Box 3000, Darwin, Northern Territory, 0801, Australia

**Keywords:** Cane toad, Frogs, PCR, Inhibition, *Entamoeba*, Invasive species, Cysts

## Abstract

The recent detection of a novel amoebozoan parasite (*Entamoeba* sp. CT1) killing invasive cane toads (*Rhinella marina)* in tropical Australia raises concerns of potential spill-over into native anuran populations. Considering the vulnerability of anuran communities globally, *Entamoeba* sp. CT1 may pose a serious threat to anuran biodiversity. Through PCR-based detection and molecular identification, we investigated the prevalence of *Entamoeba* spp. in the faeces and colon tissue of cane toads (*Rhinella marina*) and eleven native Australian frog species from a single locality in the Northern Territory. No *Entamoeba* DNA was detected in samples of native frog faeces (N = 57) or colons (N = 17). *Entamoeba* DNA was detected in 24% of 45 cane toads (95%CI 14.08–38.82). Both *E. ranarum* and *Entamoeba* sp. CT1 were present in cane toads. The failure of faecal samples to indicate *Entamoeba* spp. in infected cane toads may be due to cysts in faeces being shed intermittently, degraded before analysis, or impervious to lysis prior to DNA isolation. Our results suggest that native frogs do not carry the pathogen in an area where 20–30% of cane toads are infected with *Entamoeba* sp. CT1. We demonstrate the importance of recognising PCR inhibition prior to molecular diagnostics, and the apparent inadequacy of faecal samples for the detection of *Entamoeba* spp. in anurans.

## Highlights

• *Entamoeba* spp. were detected in cane toads, but not in faeces or colons of native frogs.
• PCR inhibition of anuran faecal samples.
• Additional purification, the presence of inhibition was reduced.

## Graphical abstract

**Figure:**
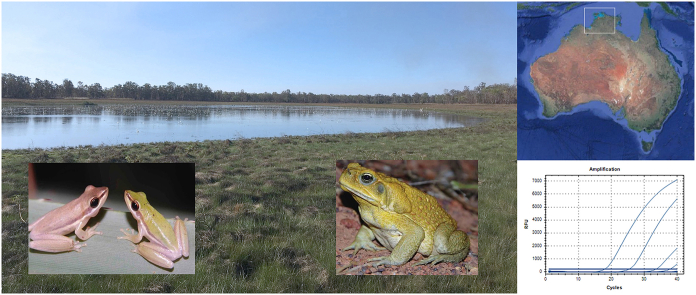

## 1 Introduction

Since its introduction into Australia in 1935 as a strategy for the control of pestiferous cane beetles *Dermolepida albohirtum* (Waterhouse, 1875), the cane toad *Rhinella marina* (Linnaeus, 1758) has become widely recognised as one of the most ecologically damaging invasive species (Sabath et al., 1981; Shine, 2018). The toad's range has expanded rapidly and now spans over 1 million km^2^ across continental Australia (Lever, 2001). The highly poisonous cane toads have direct ecological impacts upon populations of native predators through fatal toxic ingestion (Shine, 2010, 2018), and may introduce and disseminate both foreign and opportunistic native pathogens (Crowl et al., 2008; Selechnik et al., 2017).

Recently, an outbreak of lethal colitis was observed in a wild population of cane toads at the University of Sydney Tropical Ecology Research Facility (TERF) in Australia's Northern Territory (Shilton et al., 2018). Following histological analysis and environmental DNA sequencing of both sick and healthy specimens, Shilton et al. (2018) demonstrated that the causative agent was a novel amoeba belonging to the genus *Entamoeba* (hereafter ‘*Entamoeba* sp. CT1’). Although commensal amoebae (including *Entamoeba* spp.) associated with the intestinal epithelia of anurans are often detected cytologically, this is the first published link between *Entamoeba* infection and clinical gastrointestinal entamoebaiasis (Kudo, 1922). Information on the distribution of *Entamoeba* in anurans is scant in Australia, with the only reported cases involving an apparently non-pathogenic species, *Entamoeba morula*, in native frogs from Victoria and New South Wales (Raff, 1911). Considering the recency of the outbreak in the Northern Territory and that the parasitofauna of Australian anurans is poorly understood, the distribution, host-pathogen relationships and pathogenicity of *Entamoeba* sp. CT1 are still unclear.

Amphibians are declining globally, with almost a third of species currently threatened by extinction (Stuart et al., 2004). In Australia, infectious diseases such as chytridiomycosis have contributed to major losses of anuran diversity (Berger et al., 1998; Hero and Morrison, 2004; Laurance et al., 1996). If *Entamoeba* sp. CT1 is pathogenic for native Australian frogs, it may pose an emerging threat for anuran taxa already weakened by other pathogens and environmental degradation (Rohr and Raffel, 2010; Rollins-Smith, 2017; Romansic et al., 2011). Specifically, we need to know whether or not this emerging pathogen is shared between cane toads and native frogs, and if cane toads will facilitate the dissemination of *Entamoeba* sp. CT1 via spill-over or spill-back mechanisms. To answer those questions, we need to develop methods to determine the presence of *Entamoeba* spp. in frogs and toads, preferably in a non-invasive manner.

*Entamoeba* species have simple life cycles in which infective cysts are ingested by the host, pass to the large intestine and develop into trophozoites that feed on bacteria in food particles in the intestine of the host (Haque et al., 2003; Noble et al., 1989). Amoebas usually remain in the lumen of the gut, although they may favour the portion immediately adjacent to the mucosa due to appropriate pH or other microenvironmental conditions (Noble et al., 1989). Potentially pathogenic *Entamoeba* spp. are commonly detected in otherwise healthy hosts, with overt disease being uncommon; but the stimuli or predisposing factors for mural invasion are poorly understood (Faust and Guillen, 2012; Meerovitch, 1961; Ratcliffe and Geiman, 1938; Ximénez et al., 2009). Modern diagnosis of entamoebiasis in humans rests on faecal detection of either *Entamoeba* spp. antigen using antibodies specific for pathogenic species or molecular techniques such as PCR with higher sensitivity and specificity (Stark et al., 2008; Tanyuksel and Petri, 2003). Thus, application of *Entamoeba*-specific PCR in faecal samples may be a fast, simple and non-invasive means of determining the infection status of focal animals. A nonlethal method of sampling for *Entamoeba*-infection is especially desirable if a species is vulnerable or endangered.

The aim of our study was to determine the prevalence of *Entamoeba* spp. in the toad population where the outbreak occurred, and to establish whether native anurans sharing habitat with the infected toad population also host *Entamoeba* spp. (including the novel *Entamoeba* sp. CT1). We thus attempted to detect and identify *Entamoeba* present in the faeces and colon tissue of cane toads and eleven species of native Australian frogs - *Cyclorana australis* (Gray, 1842), *Limnodynastes convexiusculus* (Macleay, 1878), *Litoria bicolor* (Gray, 1842), *Litoria caerulea* (White, 1790), *Litoria dahlii* (Boulenger, 1896), *Litoria inermis* (Peters, 1867), *Litoria nasuta* (Gray, 1842), *Litoria pallida* (Davies, Martin & Watson, 1983), *Litoria rothii* (De Vis, 1884), *Litoria rubella* (Gray, 1842) and *Litoria tornieri* (Nieden, 1923) via the application of both real-time (q)PCR and conventional PCR assays, followed by DNA sequence analysis.

## 2 Materials and methods

### 2.1 Collection and processing of samples

**Fig. 1:**
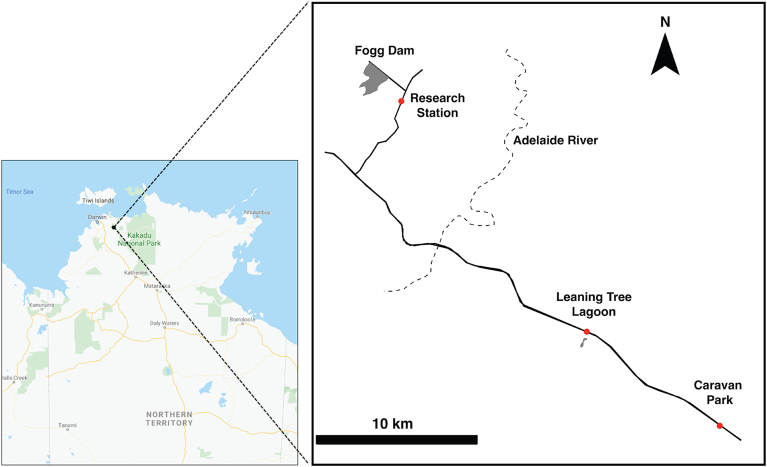
Study site location in Australia's Northern Territory (left). Map showing the Research Station where the initial amoebiasis outbreak was observed (Shilton et al., 2018); and sample collection sites Leaning Tree Lagoon and Caravan Park (right). In 2018, cane toads and native frogs were collected at Leaning Tree Lagoon. In 2020, cane toads were collected at the Caravan Park and road-killed native frogs were collected from the highway between the Research Station and Leaning Tree Lagoon. Left-hand panel image from GoogleMaps.

Our first set of samples comprised anurans that were collected on nine nights between 10 and 21 July 2018 from Leaning Tree Lagoon (12.71° S, 131.43° E, Fig. 1). We chose this 6-ha permanent water body as a study site because it acts as a dry-season refuge for both cane toads and variety of native frog species (Bleach et al., 2014; Mayer et al., 2015). Further, clinically normal cane toads from this lagoon were previously found to carry *Entamoeba* sp. CT1 (Shilton et al., 2018, 2019). Anurans were captured by hand at night along a 400 m × 30 m transect on the western shoreline of the lagoon and each individual was placed into a separate plastic bag. Animals were then returned to our research station (Fig. 1) and held in their bags overnight. The following day, bags were inspected and any faecal pellets adhering to the side of the bag were removed, preserved in 80% (v/v) ethanol and stored at room temperature. All frogs were returned to the lagoon and released at their point of capture within 24 h. Cane toads were euthanised with an overdose of pentobarbital sodium (Lethabarb, Virbac Australia) and their colons and contents dissected out, preserved in ethanol and stored at room temperature. All procedures were carried out with approval of the University of Sydney Animal Ethics Committee (permit #2018/1372) and the Northern Territory Parks and Wildlife Commission (permit #62969).

Our second set of samples collected in January 2020 included an additional 21 cane toads from a caravan park located 10 km east of Leaning Tree Lagoon (Fig. 1). The toads were returned to the laboratory where they were individually housed in 20 L plastic containers equipped with a water dish and shelter box. After each toad had defecated in its cage, we collected a sample of faeces and euthanised the toad and dissected the toad (as above) to obtain a sample of colon tissue. We also collected colons samples from 17 freshly road-killed native frogs. These frogs were retrieved from the surface of the highway between the research station and Leaning Tree Lagoon (Fig. 1), on rainy nights. We dissected colons out of these frogs and preserved them in 70% ethanol.

**Fig. 2:**
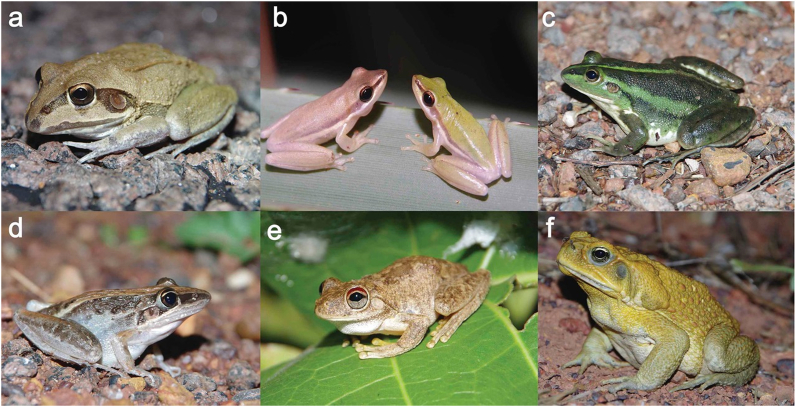
Six of the amphibian species surveyed for *Entamoeba* in this study. a) *Cyclorana australis*, b) *Litoria bicolor*, c) *Litoria dahlii*, d) *Litoria nasuta*, e) *Litoria rothii* and f) *Rhinella marina*.

A total of 173 samples were collected, consisting of 57 faecal samples (2 *Limnodynastes convexiusculus*, 8 *Litoria dahlii*, 1 *Litoria inermis*, 15 *Litoria nasuta*, 10 *Litoria pallida*, 4 *Litoria rothii*, and 17 *Litoria tornieri*) and 17 colon samples (2 *Litoria bicolor,* 6 *Litoria caerulea*, 2 *Litoria dahlia*, 5 *Litoria nasuta*, 1 *Litoria rubella* and 1 *Cyclorana australis*) from native frogs; and 28 faecal samples (passed by live toads in plastic bags overnight), 26 colon-derived faecal samples (from faeces within dissected colons) and 45 colon tissue samples from cane toads (*Rhinella marina*) (Fig. 2). All samples were placed in ethanol and stored at room temperature.

Total genomic DNA was extracted from approximately 0.05–0.25 g of each faecal sample added into tubes with glass beads and lysis buffer and disrupted using the high-speed homogeniser, FastPrep® 24 (MP Biomedicals, Australia) at 6.0 m/s for 40 s. Total DNA from 2018 was isolated using the MagMAX™ CORE Nucleic Acid Purification Kit (complex protocol) (Applied Biosystems) and those from 2020 using the Bioline ISOLATE II Faecal DNA Kit (Bioline, Australia) according to manufacturer's instructions, eluted at a final volume of 90 μL and stored at −20 °C.

### 2.2 Demonstration of PCR inhibition

All faecal samples from cane toads and at least 50% of the cane toad colon tissue and faecal samples from each anuran species were randomly selected to test for PCR inhibition. Extracted DNA samples from 2018 were further purified with either the Zymo One-Step PCR Inhibitor Removal Kit (Zymo Research, US) according to the manufacturer's instructions, or with the following modified protocol utilising the Bioline ISOLATE II Faecal DNA Kit (Bioline, Australia): Spin Filters were placed in collection tubes and prepared by adding 500 μL PCR-grade water to the filter matrix and centrifuging at 8000×*g* for 3 min 50 μL of previously eluted DNA from each sample was added into the prepared Spin Filter and centrifuged for at 8000×*g* for 1 min. The collected DNA was stored at −20 °C.

Universal bacterial 16S rDNA from both original and “purified” DNA samples was amplified to detect inhibition, with each qPCR reaction containing a total volume of 20 μL including 2 μL DNA, 10 μL 2x SensiFAST Probe No-ROX Mix (Bioline, Australia), forward and reverse universal bacteria primers [S0775, S0776], and probe [S0777] at a final concentration of 400 nM and 100 nM, respectively (Nadkarni et al., 2002). With each run, a positive control and no-template control were included to test for PCR efficiency and to detect excessive bacterial contamination. Reactions were carried out in a CFX96 Touch Real-Time PCR Detection System (BioRad, Australia) with 3 min of initial denaturation at 95 °C, followed by 40 cycles of 95 °C for 10 s and 60 °C for 30 s. Amplification was analysed between cycles 1–40 using BioRad Maestro (Version 4.1.2433.12.19) with a cycle threshold of 500 RFU. Samples with *Ct*-values > 29 (or N/A) were deemed inhibited and excluded from further analysis due to lack of amplification. “Purified” samples with successful amplification (i.e. *Ct*-value ≤ 29) were considered free from inhibition and were screened for *Entamoeba* (2.3). The remaining 2018 samples were treated with the additional purification step, and their “purified” samples were tested for inhibition using the same protocol. Original samples without inhibition evident were also screened for *Entamoeba* (2.3).

### 2.3 Molecular procedures for detection and identification of *Entamoeba* spp.

*Entamoeba* spp. were detected through the amplification of partial *Entamoeba* spp. 18 S rDNA via qPCR (Lie, 2016). The TaqMan probe and primers were designed to amplify *Entamoeba* sp. CT1 as well as *E. invadens* and *E. ranarum* only*.* Reactions were run in duplicate at a total volume of 20 μL consisting of 2 μL DNA, 10 μL 2xSensiFAST Probe No-ROX Mix (Bioline, Australia), and *Entamoeba*-specific primers and probe at concentrations described in Table 1. In each run, a no-template control and positive control were included to detect contamination and test for PCR efficiency. Reactions were carried out using CFX96 Touch Real-Time PCR Detection System (BioRad, Australia) with the same cycling conditions as for the detection of PCR inhibition (2.2). Amplification was analysed between cycles 1–40 using BioRad Maestro software with the cycle threshold defined at 500 RFU. Samples which achieved at least one *Ct*-value <38 were considered positive for *Entamoeba,* and samples with a *Ct*-value between 38 and 40 were suspected-positive. Where no amplification was detected (N/A), samples were deemed negative for *Entamoeba*.

**Table 1:** Sequences and final concentrations of primers and fluorescent probe used for the real-time PCR detection of *Entamoeba* spp.

Name	Fluorochrome	Sequence (5’→3′)	Final concentration
S0735 (Forward)		CTGCGGCTTAATTTGACT	400 nM
S0736 (Reverse)		GTTTCAGTCTCGTTCGTT	400 nM
S0737 (Probe)	5′FAM-3′BHQ1	ACTTACCAAGACCGAACATTAGAGGGA	100 nM

Selected positive and suspected-positive samples were subjected to an additional conventional PCR to produce amplicons of appropriate length for sequencing. A 387 bp *Entamoeba*-specific sequence of SSU-rDNA was amplified using primers ENTAGEN_F [S0517] (ACT TCA GGG GGA GTA TGG TCAC) and ENTAGEN_R [S0518] (CAA GAT GTC TAA GGG CAT CAC AG) (Stensvold et al., 2011). In each 30 μL reaction, 15 μL of MyTaq Red Mix (BioLine, Alexandria, NSW, Australia), 2 μL DNA, 0.5 μL of each primer, and PCR-grade water were included. With each run, a no-template control was included to detect contamination. PCRs were conducted in a Veriti Thermal Cycler (Applied Biosystems, Foster City, CA, USA), starting with initial denaturation at 95 °C for 60 s followed by 40 cycles of 95 °C for 15 s, 60 °C for 15 s, 72 °C for 30 s, and a final elongation for 5 min at 72 °C. The products were electrophoresed on 1% (w/v) agarose gel for 30 min at 100 V, and products with single bands of the expected size were purified and bi-directionally sequenced with amplification primers at Macrogen Inc. (Seoul, South Korea). After the manual removal of primers and visual inspection of chromatograms for sequence quality, the sequences were aligned and compared to known *Entamoeba* sp. CT1 and *Entamoeba ranarum* sequences stored on GenBank [accession numbers MG714920-MG714921 (Shilton et al., 2018)] using CLC Main Workbench 6.9.1 (CLC Bio, Aarhus, Denmark).

### 2.4 Statistical analyses

Basic descriptive statistics and *z*-scores were determined using Microsoft Excel 2013 (15.0.5179.1) and 95% confidence intervals for proportions/prevalences were calculated using the Wald method online with GraphPad QuickCalcs, available at https://www.graphpad.com/quickcalcs/confInterval1/.

### 2.5 Data accessibility

All SSU-rDNA sequence data generated from *Entamoeba* spp. obtained in this study have been deposited in GenBank [MN836538-MN836546]. Associated data including table with full set of diagnostic results are available from LabArchives [https://doi.org/10.25833/8fzr-wx28].

## 3 Results

### 3.1 PCR inhibition in samples of anuran faeces

Faecal and intestinal samples were assayed using pan-bacterial 16S rDNA qPCR for the presence of ubiquitous enteric bacteria. PCR inhibition was observed in all sample types across all anuran species from 2018 (Table 2). Inhibition was evident in 29 out of the 33 faecal samples from cane toads (88%), and in 31 of the 37 faecal samples from native anurans (84%). Samples of cane toad colon tissue were affected by inhibition less frequently than were faecal samples from the same species (*z* = 3.4258, *p* = 0.0003). Following additional purification protocols, the presence of inhibition was significantly reduced in faecal samples both from cane toads and from native anurans (Table 3). Samples (n = 59) from 2020 were isolated using a DNA isolation kit that included an inhibition removal step utilised above and only a single faecal sample was considered inhibited.

**Table 2:** Presence/absence of suspected PCR inhibition in anuran faecal samples, Northern Territory, Australia.

Species	Sample type	
	Faeces^a^ Inhibited*/Not inhibited	Tissue Inhibited*/Not inhibited
Cane toad (*Rhinella marina*)	29/4	11/13
Native anurans TOTAL	31/6	
Rocket frog (*Litoria nasuta*)	11/4	
Marbled frog (*Limnodynastes convexiusculus*)	1/1	
Roth's tree frog (*Litoria rothii*)	3/1	
Tornier's frog (*Litoria tornieri*)	7/2	
Dahl's aquatic frog (*Litoria dahlii*)	2/3	
The pale frog (*Litoria pallida*)	6/4	
Bumpy rocket frog (*Litoria inermis*)	1/0	

*Inhibition was suspected in samples which failed to amplify universal bacterial 16S rDNA in a qPCR assay.

a Both colon-derived faecal samples and faecal samples from cane toads (*Rhinella marina*) were grouped together as “faeces” for the purposes of this analysis.

**Table 3:** Number of anuran faecal samples from the Northern Territory, Australia with and without PCR inhibition evident, both before (Original) and after (Purified) additional purification.

Species	Treatment			
	Original Inhibited/Not inhibited	Purified^a^ Inhibited/Not inhibited	*z*-value	*p*-value*
Cane toad (*Rhinella marina*)	29/4	17/30	7.8658	<0.00001
Native anurans^b^	31/6	20/37	4.6591	<0.00001

Bolded values are statistically significant (*p* < 0.05).

Both colon-derived faecal samples and faecal samples from cane toads were grouped together as “faecal samples” for the purposes of this analysis.

*p-values indicate differences in proportion between the “original” and “purified” samples, determined by one-tailed Z tests at a 95% confidence interval.

a ”Purified” samples include those which were processed with the Zymo One Step PCR Inhibitor Removal Kit (Zymo, US) or a modified protocol utilising the final filtration column in the Bioline ISOLATE II Faecal DNA kit (Bioline, Australia).

b Native anurans included a collection of seven native Australian frog species (*Litoria nasuta, Limnodynastes convexiusculus, Litoria rothii, Litoria tornieri, Litoria dahlii, Litoria pallida and Litoria inermis*).

### 3.2 Detection and identification of *Entamoeba* in cane toads and native frogs

*Entamoeba* DNA was detected in 24% of cane toads from Northern Territory (11/45, 95% CI 14.08–38.82) in at least one type of sample. In samples from 2018, seven individual cane toads out of 24 tested (0.29, 95% CI 0.15–0.49) were found to be positive or suspected as positive for *Entamoeba* in at least one type of sample (Table 4). Five cane toads were positive in colon tissue samples, four in colon-derived faecal samples, and none in the two available samples of passed faeces. In samples from 2020, four cane toads out of 21 tested (0.19, 95% CI 0.07–0.41) were found to be positive for *Entamoeba* in at least one type of sample (Table 4). One faecal sample from 2020 was inhibited, in which the colon was *Entamoeba*-positive (late-amplifier, Ct = 37.26), while two cane toads were positive for both colon and faeces, and one cane toad was only positive using faeces for *Entamoeba* DNA.

**Table 4:** *Ct*-values^a^ from a qPCR assay for the detection of *Entamoeba* in cane toad colon, colon-derived faeces and faeces from cane toads (*Rhinella marina*) from the Northern Territory, Australia.

Cane toad ID # (year)	Sample type		
	Colon tissue	Colon-derived faeces	Faeces
28 (2018)	31.62/32.2	–	–
135 (2018)	24.93/25.92	INH	–
142 (2018)	INH	33.89/33.69	–
143 (2018)	24.13/25.04	32.36/32.59	N/A/N/A
169 (2018)	INH	37.11/N/A	N/A/N/A
170 (2018)	39.64/N/A	35.55/35.87	–
171 (2018)	22.81/23.53	N/A/N/A	–
Corr1 (2020)	37.26/N/A	–	INH
Corr2 (2020)	N/A/N/A	–	33.44/31.72
Corr7 (2020)	38.78/33.16	–	38.24/34.12
Corr10 (2020)	27.62/25.96	–	29.34/29.21

INH = sample was inhibited and was not screened for *Entamoeba*.

- = no sample available.

a *Ct*-values were analysed between 1 and 40 cycles, with the threshold defined at 500 RFU. Samples which achieved at least one *Ct*-value between 1 and 38 were considered positive for *Entamoeba*, and samples with a *Ct*-value between 38 and 40 were suspected-positive. Where no amplification was detected (N/A), samples were deemed negative for *Entamoeba*.

None of the DNA isolated from the native Australian frogs (*Cyclorana australis, Litoria bicolor, Litoria caerulea*, *Litoria dahlii*, *Litoria inermis, Litoria nasuta*, *Litoria pallida, Litoria rothii*, *Litoria rubella*, *Litoria tornieri* and *Limnodynastes convexiusculus*) returned a positive *Entamoeba* PCR result (Supplementary Table S1). Out of the 39 faecal samples collected in 2018 from native frogs, none returned positive PCR results for *Entamoeba* (0.00, 95% CI 0.00–0.11). Out of the 17 colon samples from native frogs collected in 2020, none returned positive PCR results for *Entamoeba* (0.00, 95% CI 0.00–0.22).

Amplicons with lengths of approximately 400 bp of 18 S rDNA were obtained from qPCR-positive cane toad samples. Following sequence analysis, both the novel *Entamoeba* sp. CT1 and *Entamoeba ranarum* were identified in positive cane toad samples (Table 5). Four individuals were infected with *Entamoeba* sp. CT1, two with *Entamoeba ranarum*, and one was co-infected with both species. *Entamoeba* sp. CT1 was detected in the colon-derived faeces, and *Entamoeba ranarum* in the colon tissue.

**Table 5:** Analysis of partial SSU rDNA sequence data amplified from cane toad tissue and faecal DNA samples using *Entamoeba*-specific primers.

Cane toad	Sample	Sample type	Similarity (%)^a^		*Entamoeba* species identification
			*Entamoeba* sp. CT1	Entamoeba ranarum	
28	AI	Colon tissue	100%		*Entamoeba* sp. CT1
135	DS	Colon tissue	100%		*Entamoeba* sp. CT1
142	*CJ	Colon-derived faeces	100%		*Entamoeba* sp. CT1
143	*DJ	Colon-derived faeces		100%	*Entamoeba ranarum*
	EA	Colon tissue		100%	*Entamoeba ranarum*
169	*ED	Colon-derived faeces		100%	*Entamoeba ranarum*
170	*DF	Colon-derived faeces	100%		*Entamoeba* sp. CT1
	EI	Colon tissue		100%	*Entamoeba ranarum*
171	EJ	Colon tissue	100%		*Entamoeba* sp. CT1

*Samples were purified using the Zymo One-Step PCR Inhibitor Removal Kit (Zymo, US).

a Similarity (%) was determined using CLC Main Workbench 6.9.1 (CLC Bio, Aarhus, Denmark). Sequences were aligned and compared to known *Entamoeba* sp. CT1 (GenBank accession number MG714921) and *Entamoeba ranarum* (GenBank accession number MG714920) sequences.

## 4 Discussion

Our study provides the first information on the prevalence of *Entamoeba* in cane toads (*Rhinella marina*) from Australia's Northern Territory (24.44%%, 95%CI 14.08–38.82). Congruent with findings from Shilton et al. (2018), we confirm that cane toads are hosts for both *Entamoeba ranarum* and the novel *Entamoeba* sp. CT1; and are sometimes co-infected with both species. Although *Entamoeba* sp. CT1 has only been reported once before, its sudden emergence and lethal consequences for cane toads in a region highly populated with vulnerable anuran communities make it a parasite worthy of concern and further study.

Importantly, we failed to detect *Entamoeba* spp. DNA in two out of five passed faecal samples, despite confirming the presence of *Entamoeba* spp. in colon tissue and colon-derived faeces from the same cane toads. The frogs we sampled were captured in close proximity to infected cane toads and likely were exposed to infective *Entamoeba* in the environment. Although we failed to find *Entamoeba* in the faeces of those frogs, our inability to reliably detect the parasite in faeces from two infected cane toads (those with *Entamoeba* present in their colon tissue and/or colon-derived faeces) prompted us to further test colon samples from road-killed frogs as more reliable source of diagnostic material. Our speculation that the frogs may have been infected, despite the lack of detectable *Entamoeba* DNA in their faeces, was falsified; all the colon samples were negative for *Entamoeba* spp. DNA.

*Entamoeba* spp. have a biphasic life cycle consisting of two major stages: a mobile trophozoite and an encysted form. Cysts are the common source of infection as they are more robust and able to persist in the environment, facilitating faecal-oral transmission (McConnachie, 1969). Out of the 51 described *Entamoeba* species, five lack a cyst phase (Hooshyar et al., 2015). Presence of cysts of *Entamoeba* sp. CT1 (for which the life cycle and pathogenesis remain unclear) would be consistent with Shilton et al.’s (2018) report that cytological smears from the colon of a cane toad infected with only *Entamoeba* sp. CT1 contained cysts. We failed to detect *Entamoeba* DNA in faecal samples from cane toads apparently infected with only *E. ranarum*, a species known to form cysts in anurans (Clark, 1995; Silberman et al., 1999). The absence of detectable *Entamoeba* DNA in faecal samples in our study likely reflects other factors such as inconsistent shedding ((Dobell, 1909; Kudo, 1922). Alternatively, degradation and/or DNA extraction failure has to be taken into consideration as well.

Various species of *Entamoeba* (*Entamoeba* sp. CT1, *E. ranarum, E. morula, E. ilowaiskii, E. currens, E. invadens*, *E. pyrrhogaster* and others of uncertain status) have been detected in the colonic lining of diverse reptiles and amphibians (Dobell, 1909; Hoff et al., 1984; Kudo, 1922; Shilton et al., 2018, 2019). However, cysts from infected anurans have been reported only rarely, suggestive of either an irregular or infrequent pattern of shedding (Dobell, 1909; Shilton et al., 2018). Some other protozoan parasites, such as *Giardia* spp., shed cysts in faeces only intermittently (Uchôa et al., 2017). Seasonal fluctuations in the prevalence of *Entamoeba* sp. CT1 in cane toads also suggest intermittent rather than continuous shedding of *Entamoeba* cysts (Shilton et al., 2018). The development of clinical amoebiasis in anurans is affected by factors such as season, nutritional stress and co-infection, suggesting that rates of proliferation, invasion, encystation and excretion of *Entamoeba* may be affected by the host's ability to deal with the pathogen (Faust and Guillen, 2012; Ratcliffe and Geiman, 1938; Shilton et al., 2018; Valentine and Stoskpf, 1984). Cyst production has also been found to vary with particular conditions environmental factors, for example, cyst production may be triggered by increased density of trophozoites in the colon (Faust and Guillen, 2012) or anaerobic conditions (Geiman and Ratcliffe, 1936). Hence, low or intermittent shedding rates of cysts into the faeces may explain why we failed to detect parasite DNA in the faeces of infected cane toads. In one of the passed faecal samples in question (toad #169) in which the colon tissue was unsuitable for testing, faeces from the colon tested positive but with a high C_t_ value, indicating that the negative finding from the passed faecal sample could have been stochastic due to a different sample having slightly less *Entamoeba* DNA to the point of lack of detection. This explanation is somewhat supported by the finding in the other negative passed faecal sample from an otherwise test-positive toad (#143) in which the colon tissue had more *Entamoeba* DNA (lower C_t_ value) than the colon faecal sample (higher C_t_ value), indicating that slightly different samples may have different quantities of *Entamoeba* DNA.

An alternative explanation for the lack of detectable DNA of *Entamoeba* spp. in faecal samples is due to DNA degradation during the time between defecation and collection. Closely related reptilian *E. inavdens* undergoes inducible stage conversion from trophozoite to cyst, including during axenic cell culture (Ehrenkaufer et al., 2013). Our samples were not preserved in ethanol until up to 12 h after defecation, thus *Entamoeba* may have been degraded before they were preserved with ethanol. The trophozoites of *Entamoeba* are fragile and prone to degradation outside of the host, making them difficult to detect in faeces (Tanyuksel and Petri, 2003). This hypothesis is consistent with our positive results in colon-derived faeces, which were collected immediately after euthanasia. Faecal constituents such as nucleases and/or commensal bacteria can degrade nucleic acids, resulting in poor DNA recovery (Schrader et al., 2012; Wilson, 1997).

A third possibility is that failure to rupture *Entamoeba* cysts during sample preparation and DNA extraction may have contributed to our negative results for toad faeces. The chitin-reinforced cell wall and structural properties of *Entamoeba* cysts render them resistant to chemical treatments involved in DNA isolation (Aguilar-Díaz et al., 2011; Hawash, 2014). Although we used an extraction method that is widely recommended for use in faecal samples for veterinary purposes, future work should explore the use of additional pre-processing methods to ensure cyst rupture (Hawash, 2014; Vianna et al., 2009). In addition, serial sampling of faeces produced by individuals known to be infected with *Entamoeba* would clarify the timing and quantities of cysts shedding.

The apparent inconsistency of infectious cysts in the faeces of infected toads raises an interesting ecological issue: how is the parasite transmitted to a new host, if not by ingestion of cysts in contaminated faeces? One hypothetical mechanism is via direct ingestion of tissues or intestinal contents during cannibalism, a behaviour that occurs in both the aquatic and terrestrial life-history phases of cane toads (Crossland and Shine, 2011; Hagman and Shine, 2008; Lever, 2001). However, we cannot evaluate that possibility until we know more about this system. For example, are native Australian anurans capable of becoming infected with *Entamoeba* sp. CT1 or *E. ranarum*?

Our difficulties in amplification of DNA during PCR may partially reflect the complexities of working with faeces in this respect (Schrader et al., 2012). Although there are no previous reports of the inhibition of PCR in anuran faecal samples, the presence of excreted compounds such as enzymes, complex polysaccharides, lipids, bile salts and urate in other species often interfere with *Taq* polymerase and DNA template binding (Chaturvedi et al., 2008; Kreader, 1996; Monteiro et al., 1997; Rådström et al., 2004; Schrader et al., 2012; Thornton and Passen, 2004). Our findings emphasise the importance of detecting inhibition in faecal samples prior to molecular diagnostics; whether that be through the use of pan-bacteria PCR assays (as in our study), or through the use of spiked internal positive controls (King et al., 2009).

## 5 Conclusions

For the accurate molecular detection of *Entamoeba* in cane toads, colon tissue and/or freshly collected colon contents rather than faeces collected outside of the animal's body should be used. Although our study yielded the encouraging result that all native frogs tested were negative for *Entamoeba* DNA in their faeces and colon, phenomena such as parasite degradation or low burden/shedding rates require further investigation. More generally, we need a more comprehensive understanding of the life cycle and infection dynamics of *Entamoeba* in Australian anurans, including the larval stage (tadpoles) as well as the terrestrial phase of the life cycle, to tell us whether or not the transfer of this emerging pathogen from invasive toads to native frogs is likely to pose a significant concern for conservation of the Australian anuran fauna.

## Declaration of competing interest

Authors declare no conflict of interest.
